# *Porphyromonas gingivalis* bypasses epithelial barrier and modulates fibroblastic inflammatory response in an *in vitro* 3D spheroid model

**DOI:** 10.1038/s41598-018-33267-4

**Published:** 2018-10-08

**Authors:** Isaac Maximiliano Bugueno, Fareeha Batool, Laetitia Keller, Sabine Kuchler-Bopp, Nadia Benkirane-Jessel, Olivier Huck

**Affiliations:** 1 INSERM (French National Institute of Health and Medical Research), UMR 1260, Regenerative Nanomedicine (RNM), Fédération de Médecine Translationnelle de Strasbourg (FMTS), 11 rue Humann, Strasbourg, 67000 France; 2 Université de Strasbourg (UDS), Faculté de Chirurgie-dentaire, 8 rue Sainte-Elisabeth, Strasbourg, 67000 France; 3 Hôpitaux Universitaires de Strasbourg (HUS), Department of Periodontology, 1 place de l’Hôpital, Strasbourg, 67000 France

## Abstract

*Porphyromonas gingivalis*-induced inflammatory effects are mostly investigated in monolayer cultured cells. The aim of this study was to develop a 3D spheroid model of gingiva to take into account epithelio-fibroblastic interactions. Human gingival epithelial cells (ECs) and human oral fibroblasts (FBs) were cultured by hanging drop method to generate 3D microtissue (MT) whose structure was analyzed on histological sections and the cell-to-cell interactions were observed by scanning and transmission electron microscopy (SEM and TEM). MTs were infected by *P. gingivalis* and the impact on cell death (Apaf-1, caspase-3), inflammatory markers (TNF-α, IL-6, IL-8) and extracellular matrix components (Col-IV, E-cadherin, integrin β1) was evaluated by immunohistochemistry and RT-qPCR. Results were compared to those observed *in situ* in experimental periodontitis and in human gingival biopsies. MTs exhibited a well-defined spatial organization where ECs were organized in an external cellular multilayer, while, FBs constituted the core. The infection of MT demonstrated the ability of *P. gingivalis* to bypass the epithelial barrier in order to reach the fibroblastic core and induce disorganization of the spheroid structure. An increased cell death was observed in fibroblastic core. The development of such 3D model may be useful to define the role of EC–FB interactions on periodontal host-immune response and to assess the efficacy of new therapeutics.

## Introduction

Host-bacterial interactions are crucial in the onset and development of periodontitis, a chronic inflammatory disease of infectious origin affecting tooth supporting tissues^1^. At early stage of the disease, dysbiotic flora constituted by periodontal pathogens, including *Porphyromonas gingivalis*, interacts with the epithelial barrier^2^ and induces a sustained inflammatory response^3^. Gingival epithelial cells (ECs) are the first line of host-defense challenged by oral pathogens. ECs are organized in multilayered epithelium and are challenged constantly by external pathogens at the sulcular level. Tight junctions, hemi-desmosomes and desmosomes are the key factors as they are involved in cell-to-cell and cell to extra-cellular matrix (ECM) interactions. During the establishment of the periodontal lesion, bacterial invasion or virulence factors and innate immune response activation impact such structures, leading to breakdown of the epithelial barrier and contributing to bacterial persistence within the periodontal tissues^4,5^. Sustained inflammation at the soft tissue level will be implicated in the crosstalk with underlying alveolar bone leading to its destruction^6^.

Existing *in vitro* models of monolayer cell culture have limitations as they do not allow to take into consideration the cell-to-cell interactions as cells are grown on synthetic surfaces and may form unnatural cell attachments^7^. These limitations initiate the need of using animal models that, despite being the closest to human physiological situation, are associated with ethical and technical considerations^6^. Therefore, to be able to consider the complexity of tissues or organs, 3D cell models have been developed^8–11^. 3D models have been described as mimicking more closely the natural tissues and organs physiology and phenotypes than cells grown in 2D^10,12^. The spatial proximity of cells in such a model also enables interactions between adhesion molecules and receptors maximizing cellular communication and signaling that is critical to cell function^13^. Also, cells can move, exert forces and migrate as they do *in vivo*^14^. Such 3D or multicellular models have already been used to investigate molecular mechanisms associated with infection such as for *Neisseria gonorrheae* in a 3D endometrial epithelial cell model^15^ and in pathological conditions such as cancer^16,17^. In the context of oral diseases, especially periodontitis, some models of oral mucosa were engineered to mimic the tissular organization of the multilayered epithelium and underlying connective tissue^9,18,19^. For instance, an organotypic mucosal model was developed displaying a well-organized multi-layered epithelium and underlying connective tissue characterized by collagen-embedded fibroblasts. The use of this type of a model in the context of *P. gingivalis* infection confirmed that such 3D models are more relevant than the *in vitro* 2D monolayer cultures as the cell/tissue responses observed in them appear to be closer to that of the *in vivo* models^9^.

*P. gingivalis*, a gram-negative anaerobic bacterium, is considered as a keystone pathogen as it modulates gene and protein expression compromising immune function at the periodontal level^20^. For instance, *P. gingivalis* is able to modulate inflammatory response, escape innate immunity and induce degradation of a large variety of proteins, resulting in tissue destruction. It also dampens immune response in various cell types through several mechanisms such as proteolysis as observed for NLRP3 inflammasome in endothelial cells^21^, activation of NFkB and MAPK pathways in epithelial cells and macrophages^22,23^, modulation of cell death^24^ and increase of proteases activity as observed for cathepsins^25,26^.

Mechanisms underlying intracellular invasion of epithelial cells are developed strategically by *P. gingivalis* to evade the host immune system and cause tissue damage, through its dissemination. It has been described that *P. gingivalis* influences tight junction and barrier function^27–29^. However, all mechanisms involved remain under investigation.

Therefore, the aim of this study was to develop a spheroid 3D *in vitro* model mimicking gingiva to overcome the limits of existing models and to take into account the epithelio-fibroblastic interactions. A special focus was made on *P. gingivalis* dissemination, induction of cell death and inflammation.

## Materials and Methods

### Gingival tissues

Gingival samples were obtained during periodontal surgeries (open access flap) or dental extractions from healthy patients (HP) and patients diagnosed with chronic periodontitis (CP)^30,31^. The healthy group consisted of nine patients (five men and four women; mean age, 37.8 ± 17.3 years), and the CP group consisted of eleven patients (four men and seven women; mean age, 62.4 ± 7.3 years). Samples were secured immediately in a sterile tube and stored at −80 °C until RNA extraction was carried out. All patients gave written and informed consent before enrollment. This study received approval from the Ethics Committee (French Ministry of Research, Bioethic department authorization. DC-2014-2220) and all subjects received information related to the study and gave written consent according to the Declaration of Helsinki and current French legislation.

### Bacterial culture

*P. gingivalis* strain 33277 (ATCC, Manassas, VA, USA) was cultured under strict anaerobic conditions at 37 °C in brain-heart infusion medium (Sigma, Saint-Quentin Fallavier, France) supplemented with hemin (5 mg/ml) and menadione (1 mg/ml). On the day of infection, bacteria were collected and counted as previously described^26^.

### Experimental periodontitis

**Figure 1:**
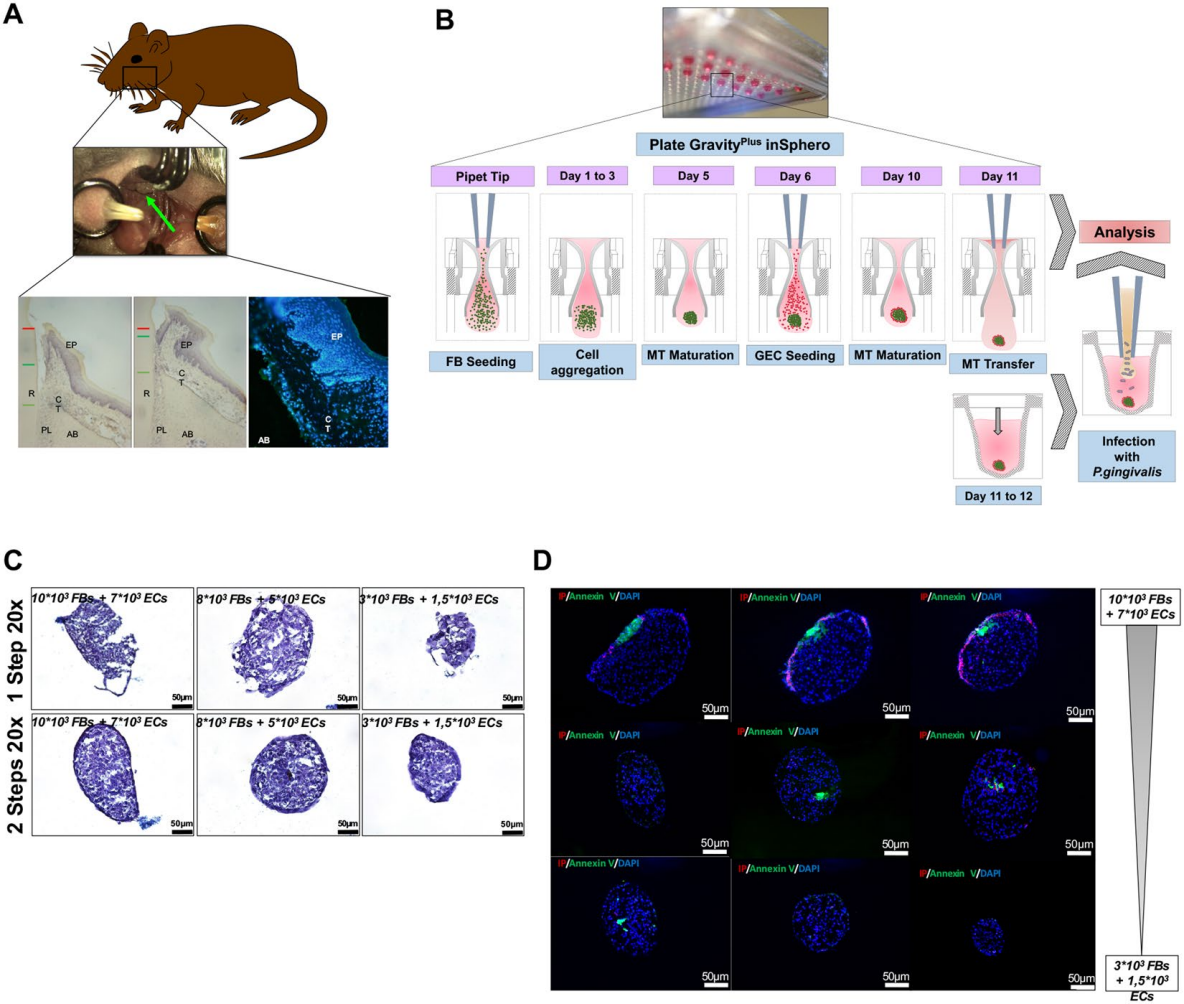
(**A**) Induction of experimental periodontitis. *P. gingivalis* infected ligatures were placed along the cervical margins on palatal sides of the maxillary first and second molars as described by Saadi-Thiers *et al*.^32^. (**B**) Microtissue spheroids formation diagram. Firstly, FBs were seeded in a droplet of culture medium for 5 days. After the formation of a spheroid, ECs were seeded over it. After 5 more days, two-cell types MTs were constituted. During all this procedure, cell culture medium was changed every 2 days. (**C**) Morphological characteristics of 1-Step and 2-Steps cell culture technique for MT spheroids. Three different cell concentrations (10 × 10^3^ FBs + 7 × 10^3^ ECs/8 × 10^3^ FBs + 5 × 10^3^ ECs/3 × 10^3^ FBs + 1.5 × 10^3^ ECs) were tested. In the 1-step procedure, both cell types were seeded simultaneously in a droplet while for the 2-steps procedure, each cell type was seeded in a time-specific manner. (**D**) Apoptosis quantification. Annexin V/IP staining of MTs at different cell concentrations showed a slight increase of cell death mainly located at the core of the MT, especially, in the biggest MT.

To induce experimental periodontitis, *P. gingivalis*-infected ligatures were placed along the cervical margins on palatal sides of the first and second maxillary molars of mice (C57/BL6, Charles River, L’Arbresle, France) (Fig. 1A). Ligatures were replaced twice a week for 30 days as described previously^32^. Mice were examined regularly to evaluate pain and stress. Moreover, their weight was monitored daily. Slides for immunofluorescence were treated as previously described by Saadi-Thiers *et al*.^33^. All experimental protocols fulfilled the authorization of the “Ministère de l’Enseignement Supérieur et de la Recherche” under the agreement number 01715.02. The Ethics Committee of Strasbourg named “Comité Régional d’Ethique en Matière d’Expérimentation Animale de Strasbourg (CREMEAS)” specifically approved this study.

### Cell culture

Human oral epithelial cells (ECs) used in this study derived from the TERT-2 OKF-6 cell line (BWH Cell Culture and Microscopy Core, Boston, MA, USA) and were cultured in KSFM culture medium (serum-free medium for keratinocytes, Gibco, PromocellTM, Aachen, Germany) and human oral fibroblasts (FBs) were isolated from gingival biopsy and cultured in RPMI 1640 medium (Life Technologies, Saint-Aubin, France). This protocol has received approval of the local Ethical Committee (DC-2014-2220). To reduce the risk of contamination, 100 units/ml of penicillin and 100 μg/ml of streptomycin were added. Both were grown at 37 °C in a humidified atmosphere with 5% CO_2_, as previously described^33^.

### Microtissues formation

To generate MTs, the hanging-drop culture method was used. MT is characterized by a 3D spheroid structure where cells are in direct contact, allowing cell-to-cell and cells-to-extracellular matrix components (ECM) interactions. This method can be used to co-culture two (or more) different cell populations to elucidate the role of cell-cell or cell-ECM interactions in a 3D environment^10,12^ (Fig. 1B). It also allows the addition of very small quantities of any biological agent or drug in the cell culture medium. Initially, 3 different concentrations of FBs (3 × 10^3^, 7 × 10^3^ and 10 × 10^3^) were cultured in suspension within a droplet of 40 μl of cells and medium in a 3D culture plate (GravityPLUS^TM^ 3D Culture, InSphero AG, Zürich, Switzerland) for 5 days, until the first true spheroid was visualized. Then, 20 μl of Defined Keratinocyte-SFM basal medium (KSFM) supplemented medium containing ECs (1.5 × 10^3^, 5 × 10^3^ and 7 × 10^3^) was added in each droplet of the suspension for 5 more days respectively (Fig. 1B,C). After testing different MT sizes, a concentration of 7 × 10^3^ FBs + 5 × 10^3^ ECs was selected for further experiments. Every 2 days, 5 μl of both cell media were added to maintain the viability and physiological conditions of the cells, preventing the drop from desiccation and consequent disaggregation.

### Infection with *P. gingivalis* and stimulation with its LPS and heat-killed *P. gingivalis*

Regarding monolayer infection, 2 × 10^5^ cells were plated in each well of a 24-wells plate. At the day of the experiment, cells were washed twice with PBS and were either infected for 24 h with *P. gingivalis* at a multiplicity of infection (MOI) of 100 or stimulated with ultrapure *P. gingivalis* LPS (1 μg/ml) (InvivoGen, San Diego, CA, USA). To ensure that effects are induced by *P. gingivalis* invasion, after 2 h of infection, the medium was removed and replaced by the same volume of bacteria free culture medium.

Regarding MT infection and stimulation, MTs were first collected from 3D culture plate. Then, MTs were infected with *P. gingivalis* at MOI 100, heat-killed *P. gingivalis* or stimulated with its LPS (1 μg/ml) (InvivoGen, San Diego, CA, USA). To inactivate bacteria, *P. gingivalis* was incubated for 15 minutes at 85 °C prior to the experiment. The MTs suspended in a 96-Well plate (CytoOne, Orsay, France) were infected for 2 to 24 h. As described for monolayer cells infection, after 2 h, the medium was replaced by a bacteria-free culture medium. To ensure *P. gingivalis* invasion, after 2 h of contact with cells or MTs, cell medium was replaced with a *P. gingivalis*-free medium. Furthermore, an antibiotic protection assay has been performed (Supplementary Figure 1) that confirmed the invasiveness of *P. gingivalis* in both cell types and MT.

### RNA isolation and reverse transcription

Total RNA from gingival samples and cells were extracted using the High Pure RNA Isolation Kit (Roche Diagnostics, Meylan, France) according to the manufacturer’s instructions. The total RNA concentration was quantified using NanoDrop 1000 (Thermofisher, Illkirch, France). 25 ng of RNA from each sample were used for Reverse transcription. Reverse transcription was performed using iScript Reverse Transcription Supermix (Biorad, Miltry-Mory, France) according to the manufacturer’s instructions.

### Quantitative real-time PCR analysis

To quantify mRNA expression, qPCR was performed on the cDNA samples. PCR amplification and analysis were performed with CFX Connect^TM^ Real-Time PCR Detection System (Biorad, Miltry-Mory, France). Amplification reactions were performed using iTAq Universal SYBR Green Supermix (Biorad, Miltry-Mory, France). Beta-actin was used as endogenous RNA control (housekeeping gene) in all samples. Primer sequences related to Bcl-2, Bax-1, Integrin β1, Apaf-1, Tnf-α, Il-6, Il-8, Col IV and β-actin were purchased from Qiagen, (Courtaboeuf Cedex, France) and ThermoFischer (Illkirch, France) (Supplementary Table 1). Expression level was calculated after normalization to the housekeeping gene expression.

### Immunofluorescence

Immunofluorescence has been performed on sections of MTs and the tissues harvested from experimental periodontitis. After embedding MTs in OCT (Tissue-Tek, Sakura Finetek, Torrance, CA, USA), 15 μm sections were obtained using cryostat (Jung, CM3000) and fixed by immersion in 4% paraformaldehyde in PBS. Sections were incubated with primary antibody against vimentin (Polyclonal goat) (1:500), E-cadherin (Monoclonal rat) (1:500), Apaf-1 (Polyclonal rabbit) (1:200), collagen IV (Polyclonal goat) (1:200), Caspase-3 (Polyclonal rabbit) (1:200), integrin β1 (1:300) and anti-*P. gingivalis* (Polyclonal rabbit) (1:5000) respectively (Supplementary Table 1) at 4 °C for 24 h. After incubation, sections were incubated with the secondary antibody for 1 h at room temperature (1:250 to 1:500 of Alexa Fluor 594 or Alexa Fluor 488 (Invitrogen, Thermofisher, Illkirch, France)). Nuclei were stained with DAPI 200 nM (Euromedex, Souffelweyersheim, France) and actin was labeled with phalloïdin Alexa Fluor™ 546 (Thermofisher, Illkirch, France). Finally, sections were mounted (Dako, Trappes, France) and observed with a fluorescence microscope (Leica DM4000B).

### Type of cell death assessment

Apoptosis and necrosis were visualized using Annexin-V-FLUOS Staining Kit according to the manufacturer’s instructions (Roche Diagnostics, Meylan, France). A solution of DAPI 200 nM (Euromedex, Souffelweyersheim, France) was added for nuclear staining. Samples were observed under an epifluorescence microscope (Leica DM 4000 B) and a digital CCD color imaging system (Microscope Digital Camera DP72; CellSens Entry, Olympus, Tokyo, Japan).

### Scanning electron microscopy (SEM)

MTs were dehydrated in a series of alcohol solutions (50, 70, 90, 100%), dried and sputter-coated for 7 to 15 min with platinum using spray coating Technics Hummer II (Technics, Alexandria, VA). Images were captured using a scanning electron microscope Leica Cambridge Stereoscan 360 FE (Leica Cambridge Co., Cambridge, UK) and the software EDS 2006 (IXRF Systems Inc., Houston, TX).

### Transmission electron microscopy (TEM)

Samples were fixed by immersion in 2.5% glutaraldehyde and 2.5% paraformaldehyde in cacodylate buffer (0.1 M, pH 7.4), and post-fixed in osmium tetroxide 1% in 0.1 M cacodylate buffer for 1 h at 4 °C, dehydrated and then treated with propylene oxide for 30 min under stirring. Samples were incorporated into Epon 812. Semi-thin sections were cut at 2 μm with ultra-microtome (Leica Ultracut UCT), stained with toluidine blue and analyzed histologically by light microscopy (Microscope Digital Camera DP72; CellSens Entry, Olympus, Tokyo, Japan). The ultra-thin sections were cut at 70 nm and contrasted with uranyl acetate and lead citrate and examined at 70 kV with an electron microscope Morgagni 268 D. Images were captured digitally by Mega View III software (Soft Imaging System).

### Statistical analysis

Statistical analysis was performed using pair-wise Anova test and post-hoc Tukey’s test. Statistical significance level was considered for *p* < 0.05. Data were analyzed using PRISM 6.0 (GraphPad, La Jolla, CA, USA). All experiments have been performed at least in triplicate (biological and technical triplicates).

## Results

### Morphological characteristics of formed MT

**Figure 2:**
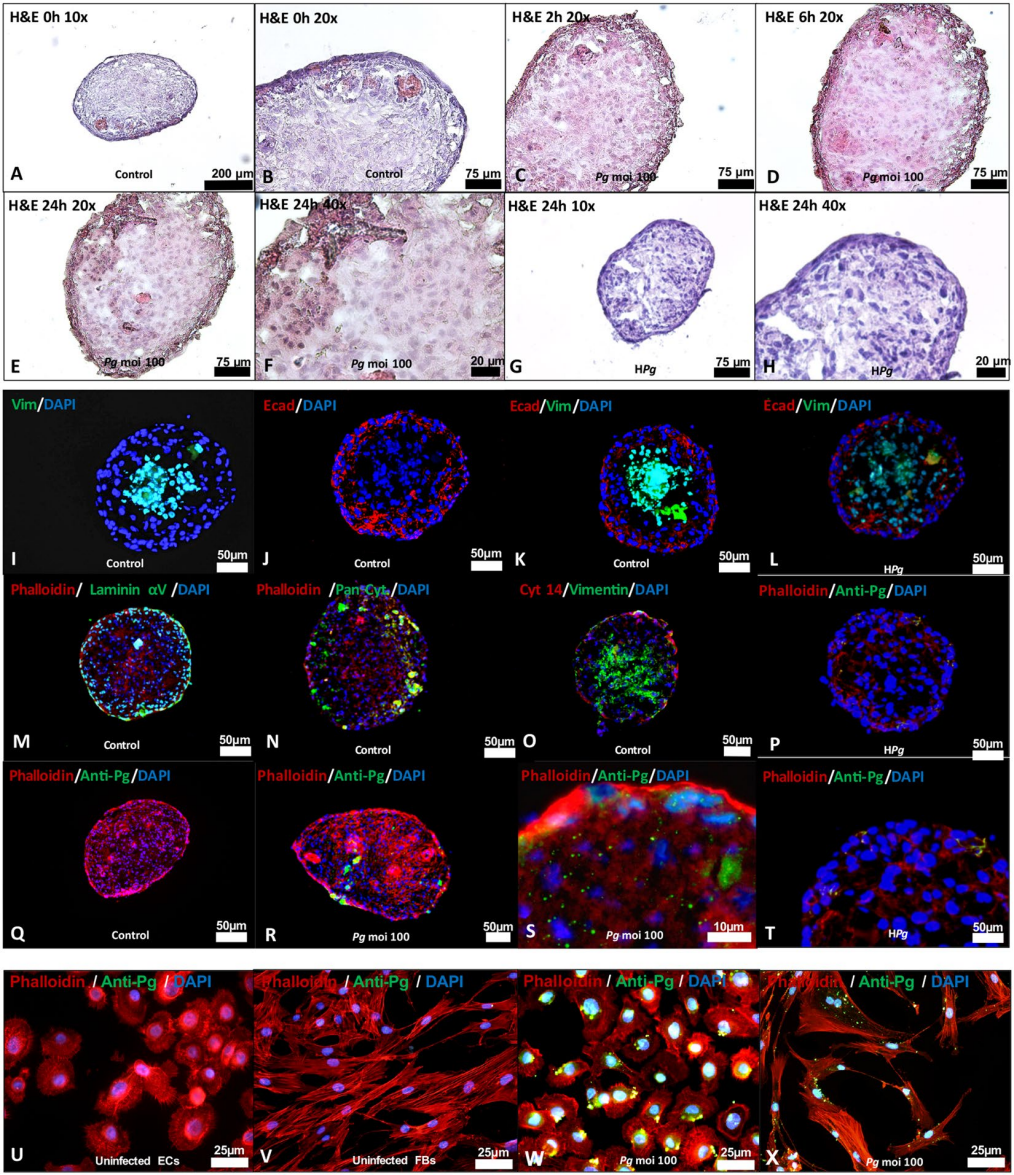
Morphological characteristics of MT spheroids mimicking gingival soft tissues. (**A**–**H**) Histological hematoxylin-eosin images under optic microscopy at 0, 2, 6 and 24 h at different magnifications (10x: **A**,**G**; 20x: **B**,**C**,**D**,**E**; 40x: **F**,**H**) of uninfected MTs (**A**,**B**), of MTs infected with *P. gingivalis* (**C**–**F**) and heat-inactivated *P. gingivalis* (**G**,**H**). (**I**–**L**) Immunofluorescence staining of uninfected MTs (**I**–**K**) and infected with heat-killed *P. gingivalis* (**L**) (Green: Vimentin, Red: E-cadherin, Blue: DAPI – nuclear staining-) (20x magnification). (**M**–**O**) Immunofluorescence staining of uninfected MTs (Green: Laminin α5; Pan Cytokeratin; Vimentin, Red: E-cadherin, Cytokeratin 14, Blue: DAPI – nuclear staining-) (20x magnification). (**P**–**T**) Immunofluorescence staining of uninfected, infected with *P. gingivalis* and heat-killed *P. gingivalis* infected MTs (Green: *P. gingivalis* immunostaining, Red: Phalloidin, Blue: DAPI – nuclear staining). (**U**–**X**) Immunofluorescence staining of uninfected, infected with *P. gingivalis* and heat-killed *P. gingivalis* infected ECs and FBs (Green: *P. gingivalis* immunostaining, Red: Phalloidin, Blue: DAPI – nuclear staining). Magnification was 20x for I-R and 40x for (**S**–**X**).

To generate gingiva-like MTs, the hanging-drop method was used to sequentially seed the FBs in the first step followed by ECs as a second layer (Fig. 1B). This sequential seeding allows the formation of well-organized MT exhibiting two distinct layers at contrary to simultaneous seeding of both cell types (Fig. 1C). Different cell concentrations for FBs and ECs were also tested (Fig. 1D) to assess impact on MT size and cell death at the core of the MT. The increase of the number of cells seeded at each step increased MT size. However, this increased size (until 200–300 μm) did not induce significant cell death at the core of the MT as observed with immunostaining with Annexin V (Fig. 1D). Formed MT exhibited a well-defined spatial organization where ECs were organized in an external cellular multilayer while FBs constituted the core of the MT (Fig. 2A,B). This distinct cellular pattern was confirmed by immunostaining with E-cadherin, pan cytokeratin and cytokeratin 14 observed at the periphery, while vimentin was mainly detected at its core (Fig. 2I–K,N,O). In addition, laminin αV was localized mainly at the ECs layer (Fig. 2M).

**Figure 3:**
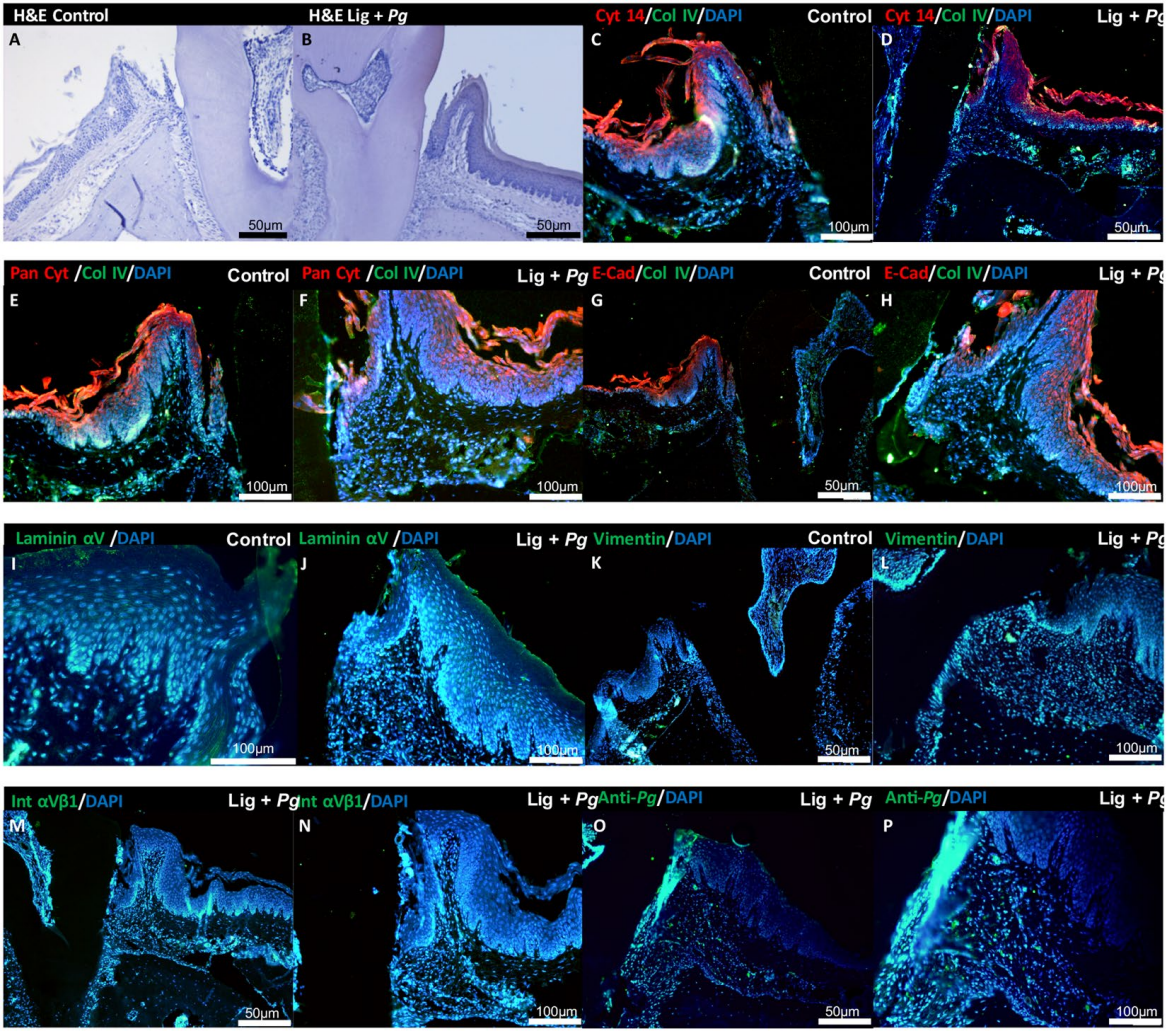
*P. gingivalis*-induced experimental periodontitis. (**A**) Histological hematoxylin-eosin staining of periodontium of non-treated site on the first molar of mice (Hematoxylin & Eosin) (10x magnification). (**B**) Morphological characteristics of periodontium of *P. gingivalis* ligature treated site on the first molar of mice (Hematoxylin & Eosin) (10x magnification). (**C**–**H**) Immunofluorescence staining for localization of Cytokeratin 14, Pan Cytokeratin and E-cadherin expression in epithelial tissue (red) and Collagen IV expression (green) in the periodontium (10x magnification). (**I**–**P**) Immunofluorescence staining for localization of Integrin β1, vimentin, laminin α5 and anti-*P. gingivalis* expression in the periodontium (green) at diseased site (10x magnification: **I**–**M** and **O**; 20x magnification: **N**,**P**). In all immunofluorescence images, nucleus has been stained by DAPI in blue.

The expression and localization of the same proteins were evaluated in an experimental periodontitis model (Fig. 3C–H). In healthy site, collagen IV and integrin β1 were localized from the epithelium-connective tissue interface until the alveolar bone border (Fig. 3C–H). Laminin αV was mainly detected at the epithelium-connective tissue interface (Fig. 3M,N). Vimentin was used as a connective tissue marker and was mainly expressed in connective tissue (Fig. 3K,L). These results confirmed the relevance of the 3D model to evaluate the mechanisms involved at the epithelial-fibroblast interface during periodontitis.

### *P. gingivalis* influenced epithelial barrier integrity

Invasion of *P. gingivalis* was evaluated by immunofluorescence in both 3D and monolayer culture. In spheroid model, invasion of MT by *P. gingivalis* was detected after 24 h (Fig. 2R,S). Interestingly, *P. gingivalis* was not only detected in the epithelial compartment but also within the fibroblastic core, demonstrating the ability of *P. gingivalis* to bypass the epithelial barrier and to disseminate within MT (Fig. 2S), hence, confirming the capability of *P. gingivalis* to invade both cell types (Fig. 2U–X) as observed *in situ* (Fig. 3O,P). Interestingly, localization of inactivated *P. gingivalis* (HPg) was not observed within the core of the MT highlighting the role of invasion in this process (Fig. 2L,P,T).

### Infection of MTs with *P. gingivalis* induced disorganization and destruction of the spheroid structure

**Figure 4:**
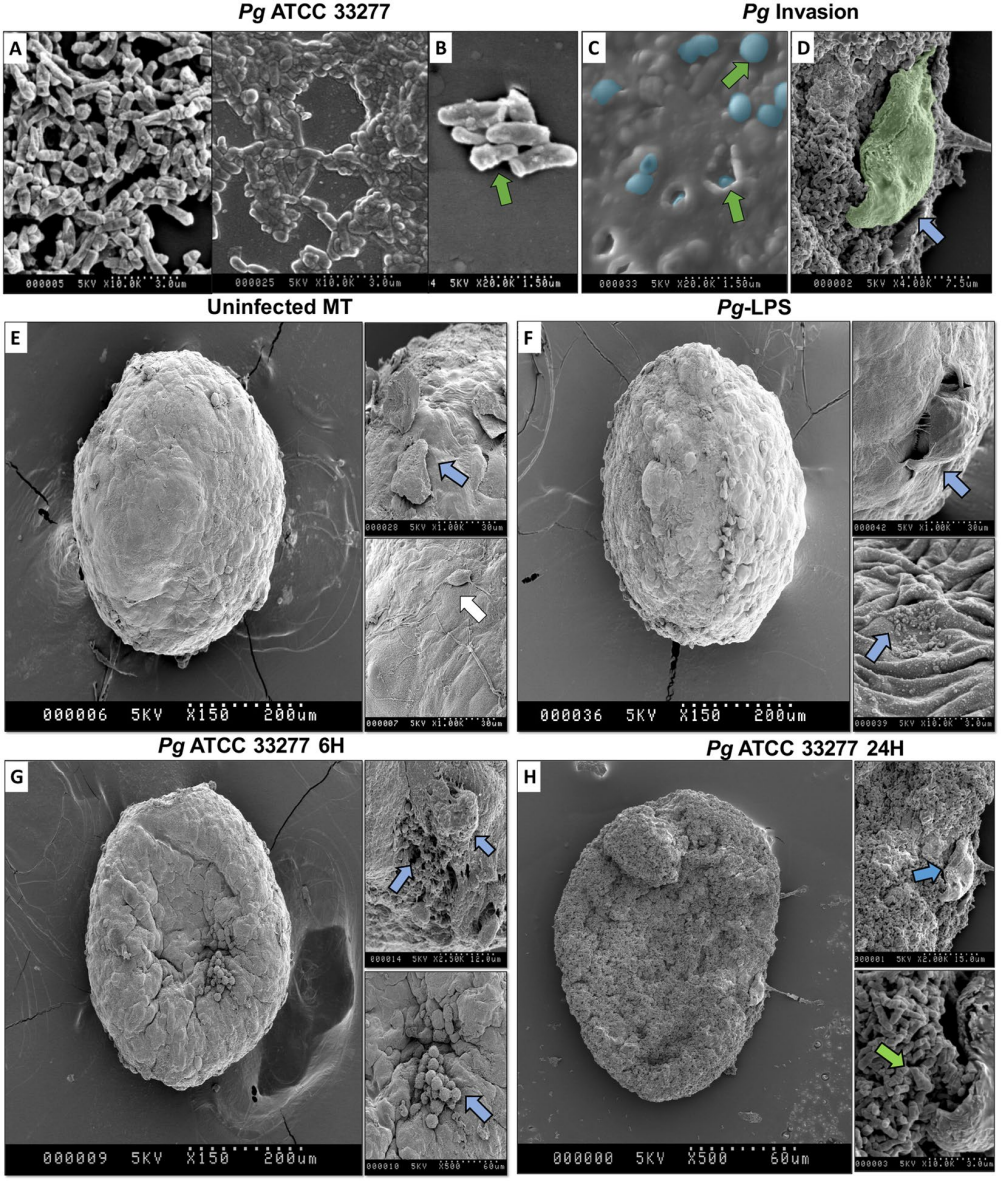
*P. gingivalis* bypasses the epithelial barrier to reach FBs located at the core of the MT leading to MT destruction. SEM imaging of (**A**,**B**) *P. gingivalis* culture, (**C**) *P. gingivalis* infection and intracellular invasion of the MT surface, (**D**) *P. gingivalis* proliferating on MT surface and EC exfoliation, (**E**) uninfected MT, (**F**) MT stimulated with LPS, (**G**) MT infected with *P. gingivalis* for 6 h and (**H**) for 24 h. Green arrows indicate *P. gingivalis* infection in MT and/or cell invasion; blue arrows indicate the disruption of epithelial barrier, multiple apoptotic cells and an acute cellular injury in all strata of the MT – EC and FB-; white arrow indicates proliferating EC at the surface of MT.

To evaluate the effect of *P. gingivalis* infection on MT structure, 3D morphometric changes were followed after infection (Fig. 4) and compared to uninfected MT at 6 and 24 h. SEM analysis showed that *P.gingivalis* was able to adhere and colonize the external epithelial surface of the MT and to be internalized (Fig. 4C). Furthermore, bacterial infection induced EC exfoliation (Fig. 4D). At 6 and 24 h, infected MT exhibited a collapsed shape associated with signs of cell injury and cell death illustrated by the detection of apoptosis (Fig. 4G,H). This observed collapsed shape seemed to be associated with the destruction of the core of the MT. Interestingly, at 24 h, infected MT exhibited signs of total destruction and some FBs could be observed even at the surface of the MT (Fig. 4H). Such destruction was not observed when MT were only stimulated with *P. gingivalis*-LPS even though structural modifications could be observed at the surface (Fig. 4F).

**Figure 5:**
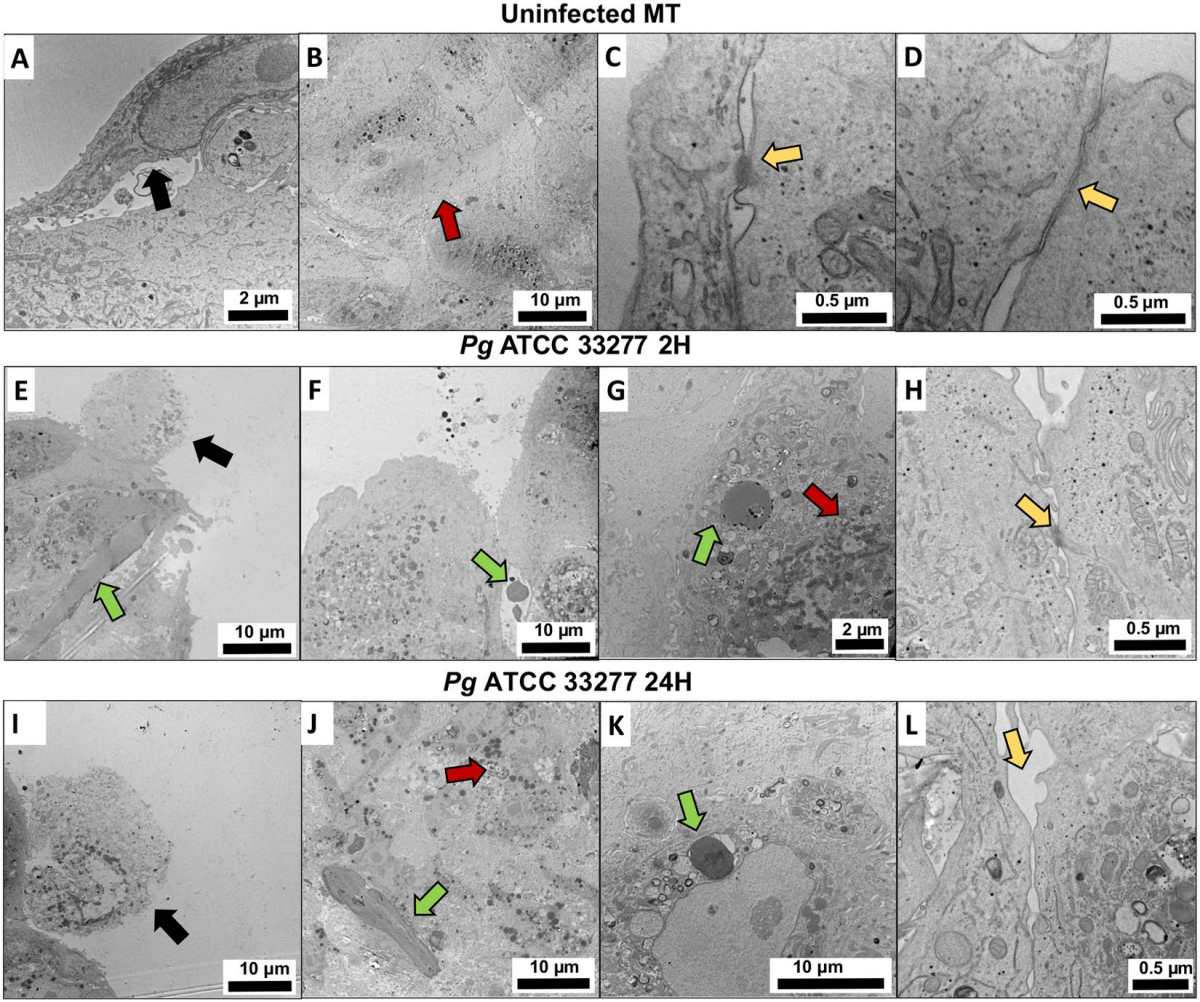
*P. gingivalis* induces fibroblastic stress and disruption of EC layer. TEM imaging of (**A**–**D**) uninfected MT, (**E**–**H**) MT infected with *P. gingivalis* for 2 h, (**M**–**O**) MT infected with *P. gingivalis* for 24 h. Green arrows indicate *P. gingivalis* bacterial infection and intercellular/intracellular invasion; black arrows indicate apoptotic cells and signs of acute cellular stress; red arrows indicate an acute cellular stress in FBs and the presence of lysosomes and apoptosis correlated with bacterial invasion; yellow arrows indicate several epithelial junctions such as desmosomes, long and short tight junctions between ECs.

**Figure 6:**
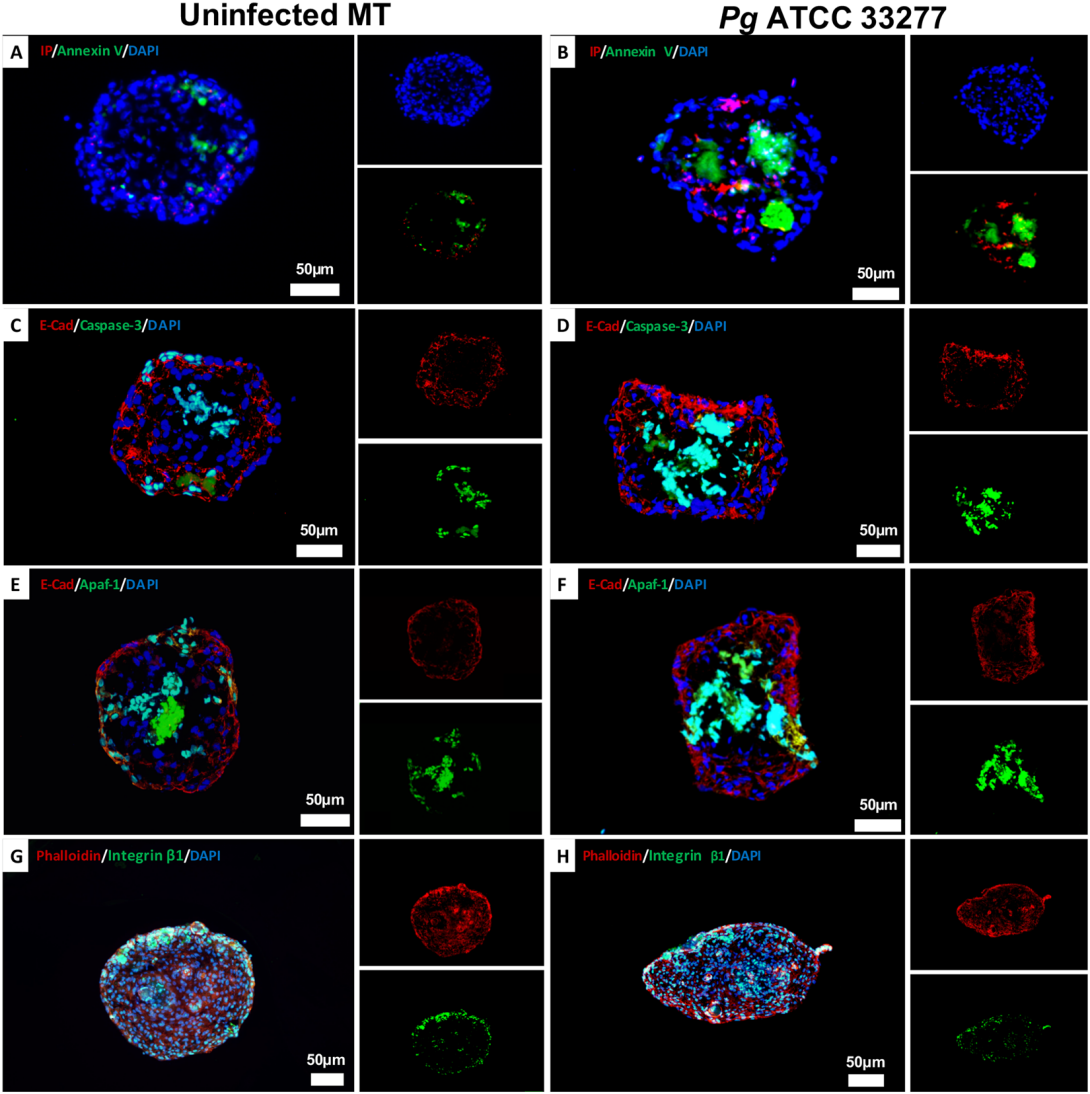
*P. gingivalis* infection induces an inflammatory response in MT correlated to an increased cell death. (**A**) Annexin V/IP fluorescence staining image in uninfected MT, (**B**) Annexin V/IP fluorescent staining images of MT infected with *P. gingivalis* for 24 h, (**C**,**D**) Immunofluorescence staining of uninfected MT (**C**) and infected with *P. gingivalis* (**D**) for 24 h (green: caspase-3, red: E-cadherin, blue: DAPI – nuclear staining), (**E**,**F**) Immunofluorescence staining of uninfected MT (**E**) and infected with *P. gingivalis* (**F**) for 24 h (green: APAF-1, red: E-cadherin, blue: DAPI – nuclear staining). (**G**,**H**) Immunofluorescence staining of uninfected MT (**G**) and infected with *P. gingivalis* (**H**) for 24 h (green: integrin β1, red: E-cadherin, blue: DAPI – nuclear staining). All images were acquired at 20x magnification.

Changes induced by infection on structural organization of the MT were also observed using TEM. Uninfected MT displayed several hallmarks of a fibroblastic core surrounded by a healthy epithelial barrier (Fig. 5A–D). Several epithelial junctions have been observed such as desmosomes, *adherens* junctions and long tight junctions between ECs (Fig. 5C,D). However, infection of MT by *P. gingivalis* induced cellular stress highlighted by the presence of apoptotic bodies correlated with bacterial invasion of ECs at 2 h (Fig. 3E–H). The effect of the infection was also visible on the integrity of the epithelial barrier illustrated by the reduction of the number of cell junctions, especially located at the most external and superficial part of ECs (Fig. 5H). At 24 h, the full disruption of the epithelial barrier, multiple apoptotic cells and an acute cellular stress in all *strata* of the MT could be observed (Fig. 5M–P). This was confirmed by immunofluorescence staining of integrin β1 which demonstrated a decreased expression at the epithelial level in response to infection (Fig. 6J,K). This pattern of expression was directly correlated to the *P. gingivalis* invasion pattern that has been shown previously by immunofluorescence and SEM (Figs 3M–O and 4).

### MT collapse correlated with *P. gingivalis*-induced apoptosis

To determine how *P. gingivalis* affects the different compartments of the MT, a focus was made on induced cell death using Annexin V/ IP staining. In comparison with the uninfected MT, at 24 h, a strong Annexin V staining was observed within the fibroblastic core (Fig. 6B) explaining the observed collapse (Figs 4 and 5). Interestingly, a differential expression of apoptosome APAF-1 and caspase-3 was simultaneously observed (Fig. 6C–H). Herein, in *P. gingivalis*-infected MT, an increased APAF-1 and caspase-3 expression was observed in FBs that emphasized the role of APAF-1 apoptosome in the activation of apoptosis. At contrary, APAF-1 detection within the ECs layer in infected MT was low (Fig. 4D,F).

### Infection with *P. gingivalis* modulated differentially the inflammation and apoptosis associated gene expression

**Figure 7:**
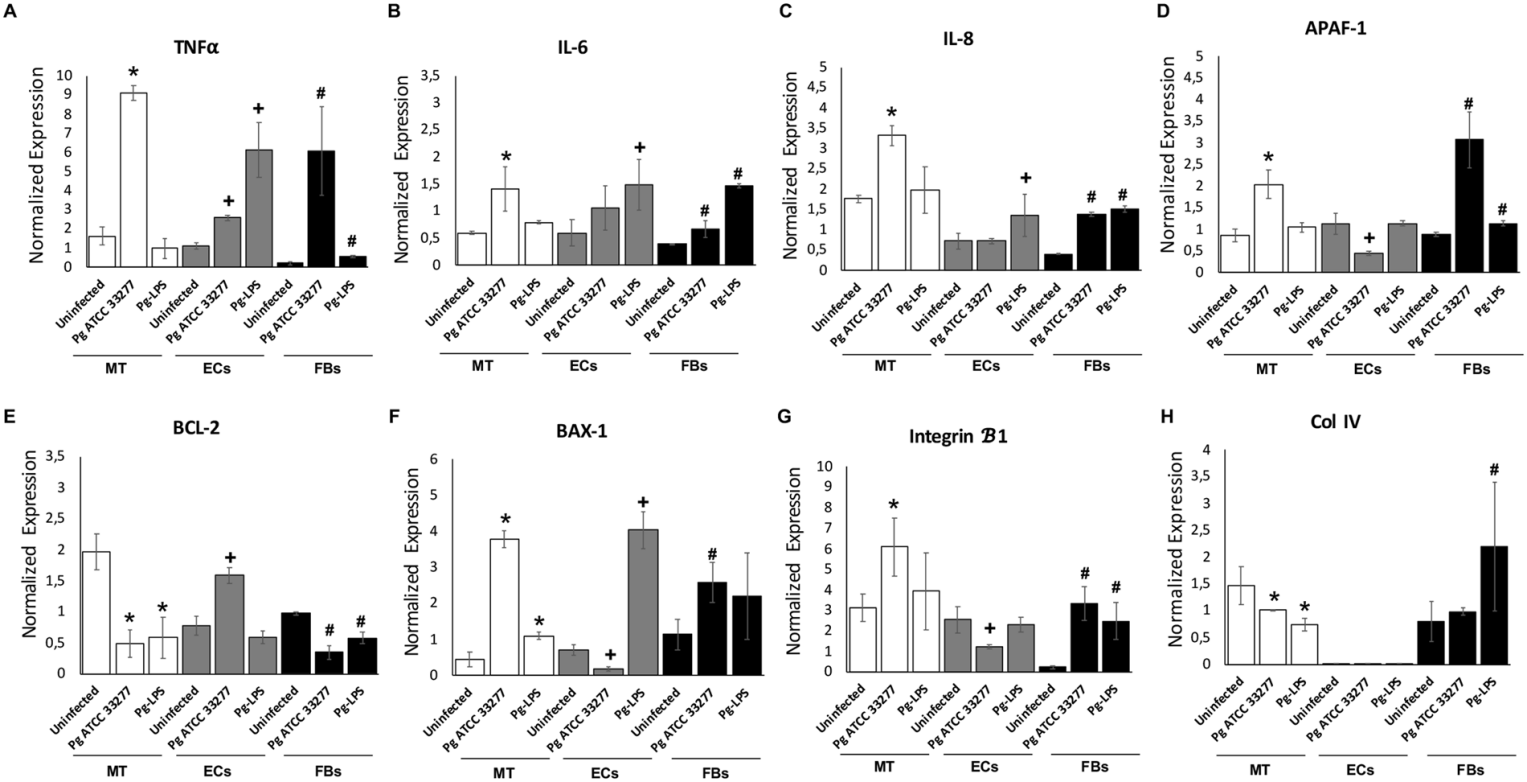
*P. gingivalis* infection induces an inflammatory and pro-apoptotic cell death response in MT correlated to increased gene expression. (**A**–**F**) Gene expression of inflammatory and pro-apoptotic cell death response related genes: TNF-α, IL-6, IL-8, Bax-1, Bcl-2 and Apaf-1 in MT; monolayer cell culture of ECs and FBs infected with *P. gingivalis* at MOI 100 for 24 h; (**G**,**H**) Gene expression of integrin β1 and collagen IV. *Differences between infected or stimulated MT vs control (*p* < 0.05). ^+^Differences between infected or stimulated ECs vs control (*p* < 0.05). ^#^Differences between infected or stimulated FBs vs control (*p* < 0.05).

To evaluate the pattern of gene expression associated with inflammation, apoptosis and epithelial integrity, mRNA expression in MT in response to *P. gingivalis* infection was measured and compared to that of monolayer cell culture. Regarding inflammation, infection of MT increased TNF-α, IL-6 and IL-8 expression (Fig. 7A–C). Interestingly, same trend of results was observed in monolayer cell cultures. Effect on apoptosis has also been evaluated focusing on Bcl-2/Bax-1 and Apaf-1. In ECs, infection with *P. gingivalis* displayed anti-apoptotic effects through a decrease of Bax-1 and Apaf-1 and an increase of anti-apoptotic Bcl-2 expression in EC (Fig. 7D–F). In FBs, *P. gingivalis* infection induced opposite effects as it increased the expression of pro-apoptotic markers (Fig. 7D–F). The gene expression of MT was closer to that of FBs. The breakdown of the epithelial barrier was also confirmed as mRNA expression of integrin-β1 was significantly decreased by infection (Fig. 7G).

**Figure 8:**
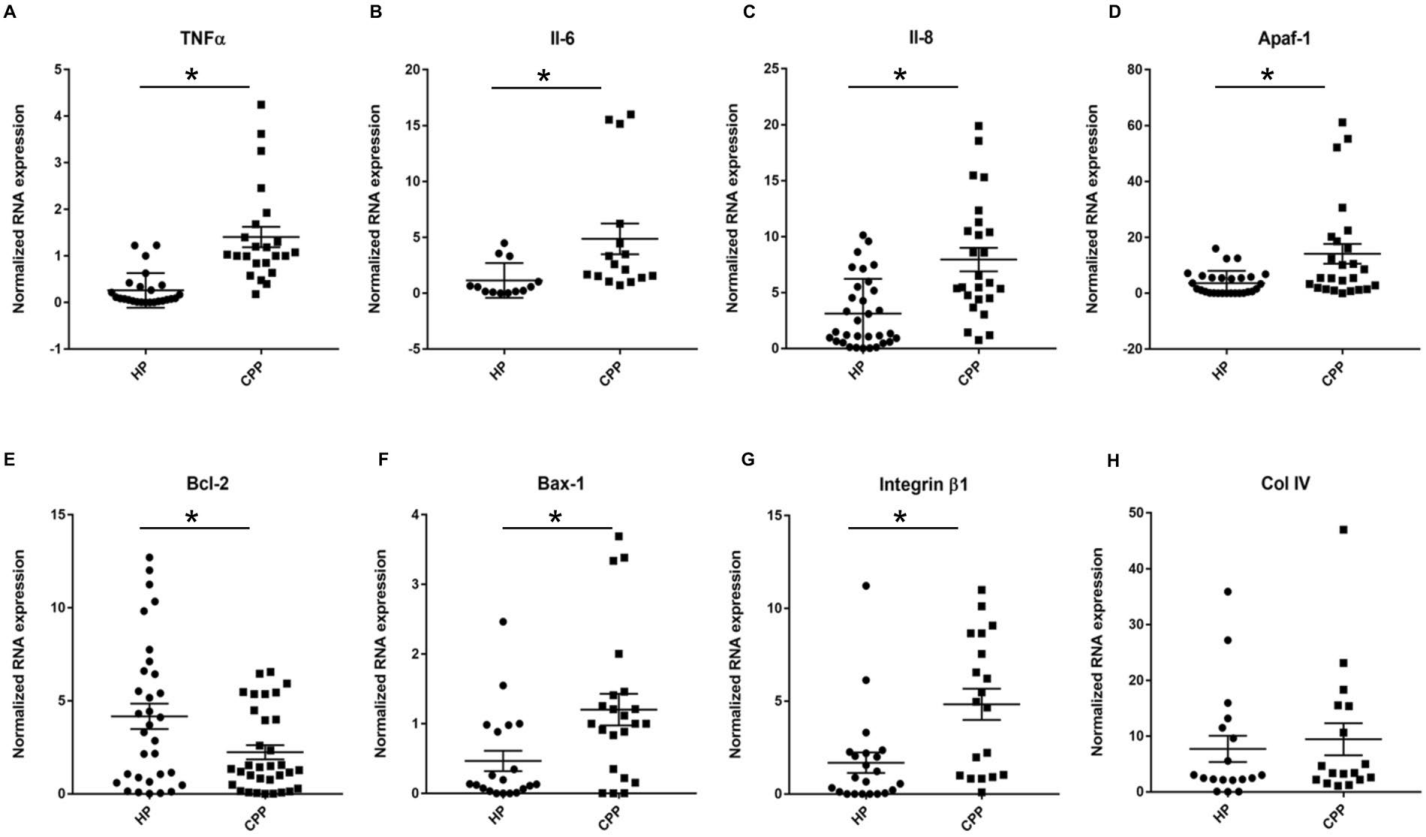
Inflammatory and pro-apoptotic cell death related gene expression is increased in chronic periodontitis samples. (**A**–**F**) Gene expression of inflammatory and pro-apoptotic cell death response related genes: TNF-α, IL-6, IL-8, Apaf-1, Bcl-2 and Bax-1 from mRNA extracted from chronic periodontitis patients (CPP) (11 patients) and healthy patients (HP) (9 patients); (**G**,**H**) Gene expression of integrin β1 and collagen IV from mRNA extracted from chronic periodontitis patients (CPP) (11 patients) and healthy patients (HP) (9 patients). *Differences between CPP and HP samples (*p* < 0.05).

To evaluate if the mRNA expression pattern observed in infected MT was comparable to that expressed in diseased tissues, mRNA expression of the same genes was evaluated in human gingival tissues harvested from healthy (H) and chronic periodontitis (CP) patients (Fig. 8). Analysis revealed that the expression of TNF-α, IL-6, IL-8, Bax-1 and Apaf-1 mRNAs was significantly increased in the CP group (TNF-α, 4.7-folds; IL-6, 3.85-folds; IL-8, 2.5-folds; Bax-1, 3-folds; Apaf-1, 3.9-folds) (*p* < 0.05) (Fig. 8A–D,F) while the anti-apoptotic Bcl-2 expression was decreased (1.9-folds) (Fig. 8E). As observed in MT, mRNA expression of integrin β1 was also increased significantly in CP (Fig. 8G). These results confirmed that the modulation of mRNA expression by *P. gingivalis* in MT is similar to the one observed in human diseased tissues.

## Discussion

In this study, we designed a spheroid 3D *in vitro* model of gingival tissue and assessed the destructive effects associated with *P. gingivalis* infection. The well-organized 3D structure of the synthesized MT mimicked the epithelial-fibroblastic barrier and allowed investigation of the *P. gingivalis*-induced effects associated with bacterial invasion and to focus on the epithelial barrier disruption and inflammation.

It has already been described that the synthesis of 3D MT using hanging-drop method is a reliable technique to generate homogenous organs or tissue-like structures at low-cost without requiring specific growth factors or complicated technical procedures. This type of spheroid model has been used previously to determine the physiopathological processes associated with diseases such as cancers, cellular organization during embryogenesis, drug testing and also as potential therapeutics such as demonstrated in the context of dental pulp regeneration^10,17,34–36^. The use of this model is of interest to evaluate the effects associated with the infection by *P. gingivalis* or by other periodontal pathogens while taking into account the epithelio-fibroblastic interactions. This coupling in 3D maximizes cell-to-cell communication and signaling that is critical to cell function. Furthermore, the phenotype or function of cells grown in 3D is more complex and closer to the functions of human tissues compared to the cells grown in 2D^37^. Hypoxia can be a physiologically relevant phenomenon considered as a major characteristic of 3D microenvironments both *in vitro* and *in vivo*. Oxygen concentration in 3D tissues depends on the balance between oxygen delivery and consumption^38^. In cancer models, hypoxic conditions need to be included in the 3D scaffold design and this can be done by strictly regulating the size of the MT. It has been shown that oxygen can diffuse across 100–200 μm of tissue thickness and it is generally desirable to maintain the optimal aggregate size of approximately 250 μm to prevent hypoxia^39^. In this study, the number of cells seeded was standardized to an ideal size for which the cells in the core of the uninfected MT remained healthy and unaffected by hypoxia. However, a modification of the protocol of MT synthesis may be done, through an increase in the number of the cells seeded, to evaluate the effect of hypoxia on cells response to infection as this phenomenon was demonstrated to be deleterious to oral health^40^. Such 3D model may also be interesting in the future to evaluate the effects of anti-infectious or anti-inflammatory drugs while considering tissular penetration.

Several other 3D models of oral mucosa have already been developed^9,41–43^. Most of them are collagen-based oral mucosal models (OMM) and displayed efficiently the characteristics of para-keratinized tissue. Interestingly, the feasibility of combining such engineered mucosa with engineered alveolar bone was also established^43^. The use of such models confirmed the need of 3D models to investigate inflammatory processes and also host-response to pathogens, such as *P. gingivalis*, as differential effects between 3D and 2D models were highlighted, especially, regarding inflammatory cytokines (IL-8, IL-1α) secretion^9^. In our 3D MT model, same discrepancies related to the mRNA expression of inflammatory markers were also observed in comparison with monolayer cultures. Furthermore, the gene expression of the non-infected and *P. gingivalis*-infected MT was similar to that measured in healthy and diseased gingival tissues respectively, confirming the similarity between MT and gingival tissue. It should be mentioned that one limitation of the MT model is the absence of keratinization that could be obtained and controlled in OMM after exposure of the cell layers to air^9,43,44^.

In MT, the external localization of ECs mimics the epithelial barrier, a key element of the host-defense and innate immune response due to its implication in host-bacterial crosstalk^45^. At the junctional epithelium, ECs are connected to each other by a variety of specialized transmembrane proteins including tight junctions, *adherens* junctions, gap junctions and desmosomes^27,28^ that constitute a thin structure. Adhesion structures are the key to maintain epithelial integrity and are also involved in the coordination of several signaling and trafficking molecules, thereby, regulating cell differentiation, proliferation, and polarity^46–48^. In our 3D model, ECs were characterized by the presence of E-cadherin observable at the periphery of the MT and localized at the epithelial level, confirming an architecture similar to that of the junctional epithelium. Following *P. gingivalis* infection, we observed *in vivo* and in MT, the bacterial ability to invade ECs, as demonstrated previously, and also to bypass the epithelial barrier to reach the underlying tissue. Invasion is a rapid process and is accompanied by cytoskeletal modifications, calcium ion fluxes, modulation of MAP kinase and apoptotic pathways, and downregulation of IL-8 expression^49,50^ contributing *in vivo* to bacterial persistence and progression of the chronic aspects of periodontitis^51^. Several studies demonstrated that *P. gingivalis* induces proteolysis of adhesion structure components and modulates related gene expression^52,53^. Here, we focused on integrins due to their involvement in binding and invasion of *P. gingivalis* as demonstrated in several cell types^54,55^. In this study, *P. gingivalis* strain 33277 was used as it is a well-described invasive strain exhibiting several virulence factors such as fimbriae and our laboratory has significant experience using this strain^24,56,57^. Future studies should explore the precise role of each virulence factor in the invasion process and inflammatory response observed in MT model.

Modulation of apoptosis is a key feature of innate immune response subversion elicited by *P. gingivalis*, especially at the epithelial level^33,58^. In MT, the expression of apoptotic markers (caspase-3, Bax-1/Bcl-2, Apaf-1) was differentially measured according to cell type as observed previously^33^. In MT, APAF-1 related apoptosome was mostly expressed in fibroblastic core and its expression within epithelial layer was decreased following *P. gingivalis* infection. The inhibition of apoptosis in ECs is a well-described mechanism involved in *P. gingivalis* pathogenesis. Several studies demonstrated the targeting of cells with a rapid turnover, such as junctional epithelial cells, by *P. gingivalis*^59^ and its ability to decrease epithelial apoptosis. Therefore, the use of MT model will be useful to evaluate indirect impact of *P. gingivalis* on connective tissue following ECs stimulation.

In conclusion, this 3D *in vitro* model of gingival tissue may be used to analyze pathophysiological processes involved in periodontitis especially molecular mechanisms related to either innate immune response, role of bacterial virulence factors occurring at the epithelium-connective tissue interface and therapeutic properties of potential anti-inflammatory or anti-infective drugs. The potential of adding a third cell type at the core of the MT, such as osteoblasts, may be of interest to investigate the influence of soft tissue infection and inflammation on soft tissue-bone crosstalk.

## Electronic supplementary material

Supplementary Information
