# Supplementary material for: Porphyromonas gingivalis bypasses epithelial barrier and modulates fibroblastic inflammatory response in an in vitro 3D spheroid model
Source: Sci Rep. 2018 Oct 8;8:14914. doi: 10.1038/s41598-018-33267-4 (PMC6175856; doi:10.1038/s41598-018-33267-4)
Supplement: Supplementary file 1 — Supplementary Information [file 41598_2018_33267_MOESM1_ESM.pdf]

Porphyromonas gingivalis **bypasses epithelial barrier and modulates fibroblastic inflammatory response in an** ***in vitro*** **3D spheroid model**

Isaac Maximiliano Bugueno, Fareeha Batool, Laetitia Keller, Sabine Kuchler-Bopp, Nadia Benkirane-Jessel, Olivier Huck

**A) Primers**		
**Gene**	**Primer Sequence 5' – 3' (Forward)**	**Primer Sequence 5' – 3' (Reverse)**
*Bcl-2 **	--	--
*Bax-1 **	--	--
*Integrin ℬ-1 **	--	--
Apaf-1 ^	3'-GTCTGCTGATGGTGCAAGGA-5'	5'-GATGGCCCGTGTGGATTTC-3'
*Tnf-α^*	3'-TCTTCTCCTTCCTGATCGTG-5'	5'-GAAGATGATCTGACTGCCTG-3'
Il-6 ^	3'-AATCATCACTGGTCTTTTGGAG-5'	5'-GCATTTGTGGTTGGGTCA-3'
Il-8 ^	3'-GAACCATCTCACTGTGTGTAAA-5'	5'-CACTCCTTGGCAAAACTG-3'
ℬ-actin ^	3'-TTGGCAATGAGCGGTT-5'	5'AGTTGAAGGTAGTTTCGTGGAT-3'
Col IV	3'-ATGGGGCCCCGGCTCAGC-5'	5'-ATCCTCTTTCACCTTTCAATAGC-3'

**B) Antibodies**	
**Target**	**Characteristics**
CYTOKERATIN 14	Santa Cruz Biotechnologies<br>Goat Polyclonal
PAN CYTOKERATIN (AE1.AE3)	Santa Cruz Biotechnologies<br>Goat Polyclonal
INTEGRIN αVℬ-1	Chemicon International<br>Alexa Fluor™ 488
LAMININ αV	Santa Cruz Biotechnologies<br>Goat Polyclonal
VIMENTIN	Abcam<br>Rabbit polyclonal
E-CADHERIN	Takara Bio Europe<br>Rat monoclonal
COLLAGEN IV	Santa Cruz Biotechnologies<br>Goat Polyclonal
ANTI-*Pg*	Provided for Dr Richard Lamont, USA<br>Rabbit polyclonal
APAF-1	Thermofischer<br>Rabbit polyclonal
CASPASE-3	Thermofischer<br>Rabbit polyclonal
PHALLOÏDIN	Thermofisher<br>Alexa Fluor™ 594

Supplementary Table 1. (A) Sequence of primers used for real-time quantitative RT-qPCR;

(*) Primer sequences were not provided by the manufacturer. (B) Antibodies used for immunofluorescence staining.

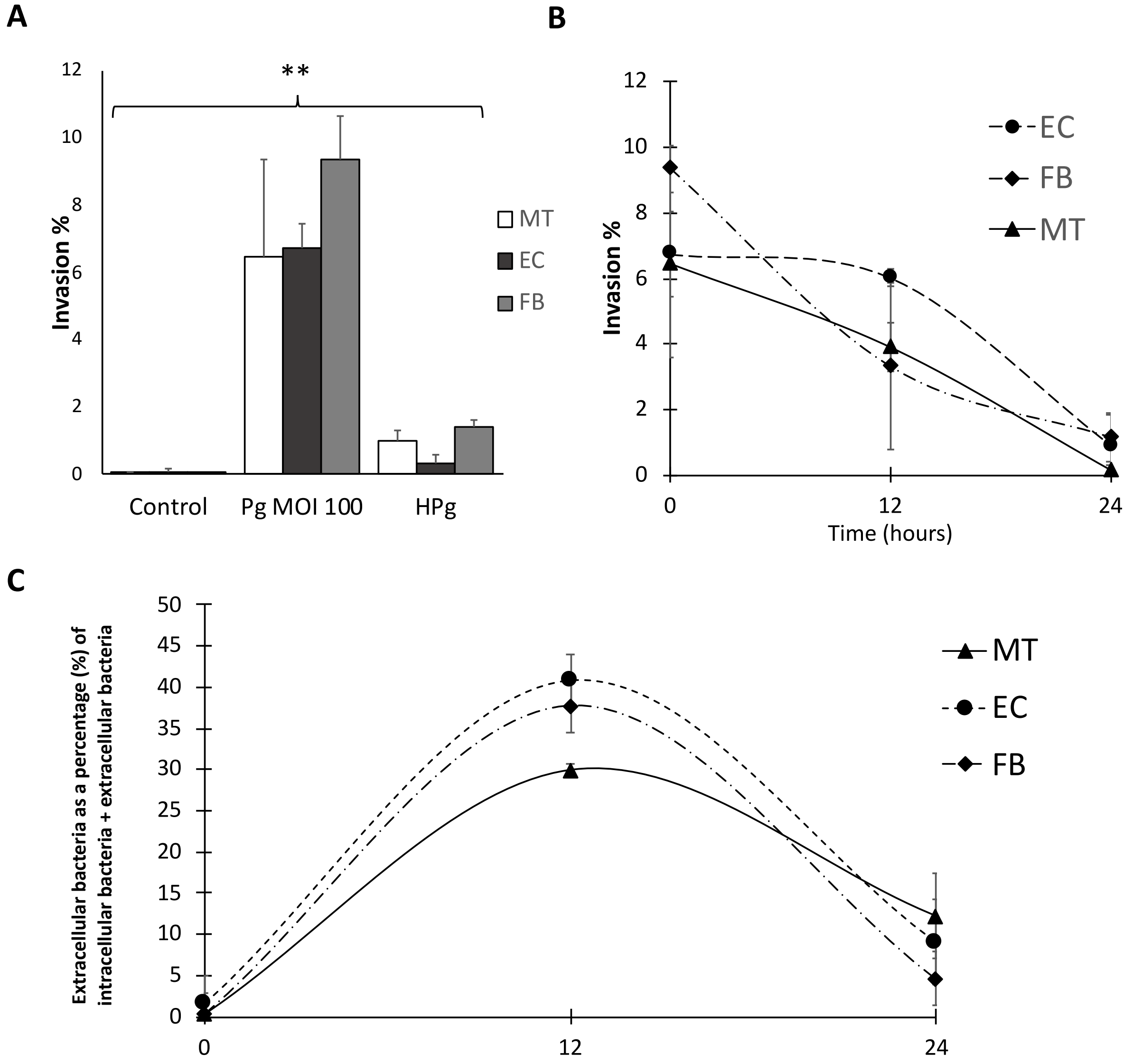

Supplementary Figure 1. Percentage of invasion evaluated by antibiotic protection assay. After monolayers ECs, FBs and MT culture, infection with *P. gingivalis* ATCC 33277 was performed at a multiplicity of infection (MOI) of 100 (for monolayers) or $1*10^7$ bacteria/ml (for MT; determined to be an equivalent of MOI 100 for a total of 10 MT; 1 MT = ~$1*10^4$ cells) suspended in the KSFM culture medium (serum-free medium for keratinocytes, Gibco, PromocellTM, Aachen, Germany) for ECs, in RPMI 1640 medium (Life Technologies, Saint-

Aubin, France) for FBs and in KSFM/RPMI medium (1:1) for MT. After 1.5 (monolayers) or 2h (MT) of infection at 37°C, monolayers and MT were washed three times with PBS to remove non-adherent and external bacteria, then metronidazole (200μg/mL) was added for 1h to kill the external bacteria. Intracellular bacteria were released by osmotic lysis using sterile distillated water and scraping the cells. Just after lysis, the number of bacteria was quantified by TaqMan Real-Time PCR (Suzuki et al., 2005). Primer sequences used were: universal 16S rRNA (Forward, TCC TAC GGG AGG CAG CAG T, Reverse, GGA CTA CCA GGG TAT CTA ATC CTG TT), *P. gingivalis* (Forward, CCT ACG TGT ACG GAC AGA GCT ATA, Reverse, AGG ATC GCT CAG CGT AGC ATT). The number of intracellular bacteria is expressed as the percentage of the infecting inoculum (MOI 100). Graphs show mean and SD of three independent experiments performed in triplicate, $**p < 0.01$.

(A) Invasion of monolayer ECs and FBs, and MT infected with *P. gingivalis* ATCC 33277 and heat-inactivated *P.gingivalis*. (B) The line charts show invasion of monolayer ECs and FBs, and MT, cultured using EC + FB, infected with *P. gingivalis* ATCC 33277 at 0, 12 and 24 hours after antibiotic treatment. (C) The number of bacteria released from the cells on monolayers or MT, which is presented as the percentage of extracellular bacteria of the intracellular + extracellular bacteria estimated by TaqMan Real-Time PCR quantification.

Reference:

Suzuki, N., Yoshida, A. & Nakano, Y. Quantitative analysis of multi-species oral biofilms by TaqMan Real-Time PCR. *Clin. Med. Res.* **3**, 176-185 (2005).
